# Interleukin-8 does not influence proliferation of the SGC7901 gastric cancer cell line

**DOI:** 10.3892/ol.2014.2531

**Published:** 2014-09-12

**Authors:** JUN SHI, PIN-KANG WEI

**Affiliations:** Department of Traditional Chinese Medicine, Shanghai Changzheng Hospital, The Second Military Medical University, Shanghai 200003, P.R. China

**Keywords:** interleukin-8, proliferation, gastric cancer, proliferating cell nuclear antigen, SGC7901 gastric cancer cell line

## Abstract

Interleukin-8 (IL-8), an important inflammatory factor, is induced by *Helicobacter pylori* infection and is clearly associated with gastric cancer. IL-8 levels have been revealed to correlate significantly with the adhesion, migration and invasion of gastric cancer cells. However, whether IL-8 influences cell proliferation in gastric cancer remains unclear. In the present study, the effect of IL-8 on the proliferation of the SGC7901 human gastric cancer cell line was investigated. SGC7901 cells were exposed to various concentrations of IL-8 (0, 0.2, 0.5, 0.8 and 1 ng/ml) for one to seven days. Cell proliferation was detected by Cell Counting Kit-8 assay. In addition, proliferating cell nuclear antigen (PCNA) protein and mRNA levels were measured by immunofluorescence, western blotting and quantitative polymerase chain reaction. Following exposure of SGC7901 cells to the various concentrations of IL-8, no significant changes in terms of cell proliferation were identified. However, IL-8 was shown to regulate PCNA protein and mRNA expression levels; at a concentration of 0.8 ng/ml, IL-8 significantly elevated the PCNA protein and mRNA expression levels, whereas IL-8 significantly inhibited these levels at other concentrations, compared with no treatment. In conclusion, IL-8 does not affect the proliferation of SGC7901 cells. However, IL-8 dosage was associated with PCNA protein and mRNA expression levels.

## Introduction

Gastric cancer was the leading cause of mortality from gastrointestinal malignancy worldwide in 2012 (1,2). A number of pathways and genes have been implicated in the development of gastric cancer. Interleukin-8 (IL-8), a member of the neutrophil-specific CXC subfamily of chemokines, is important not only in leukocyte chemotaxis, inflammatory responses and infectious diseases (3), but also in the proliferation, invasion and migration of endothelial cells (4,5). Previous studies suggested that solid tumors, including those of prostate, breast and ovarian cancer, express IL-8 (6,7). IL-8, an autocrine growth factor, promotes tumor growth, tissue invasion and metastasis (5). Upregulation of IL-8 occurs in gastric cancer (8) and has been associated with the adhesion, migration and invasion of human gastric cancer cells (9). Recent studies have demonstrated that *Helicobacter pylori* (Hp) infection causes extensive gastric epithelial cell inflammation, which may result in atrophic gastritis, intestinal metaplasia and even gastric adenocarcinoma (10–15). Furthermore, IL-8 levels are higher in Hp-infected gastric tissue than in Hp-negative tissue (16).

Overexpression of IL-8 has been associated with invasion and metastasis in gastric cancer; however, the association between IL-8 and gastric cancer cell proliferation remains unclear. The present study evaluated whether IL-8 affects the proliferation of the SGC7901 gastric cancer cell line, and investigated the effect of IL-8 on the expression levels of proliferating cell nuclear antigen (PCNA) protein and mRNA.

## Materials and methods

### Cell culture

The SGC7901 human gastric cancer cell strain was purchased from the Cell Bank of Type Culture Collection of Chinese Academy of Sciences (Shanghai, China), and the cells were inoculated in RPMI-1640 medium (Genom Biopharmaceutical Technology Co., Ltd., Hangzhou, China), supplemented with 10% fetal bovine serum (Zhejiang Tianhang Biological Technology Co., Ltd., Hangzhou, China), 1% penicillin/streptomycin and 1% L-glutamine. The cells were maintained at 37°C in a humidified chamber containing 5% CO_2_.

### Cell grouping and drug treatment

IL-8 stock solution (Sigma-Aldrich, St. Louis, MO, USA) was added to each well at a predetermined concentration. Therefore, based on IL-8 dosage, the following five groups were experimentally maintained: 0, 0.2, 0.5, 0.8 and 1 ng/ml groups.

### Cell proliferation assay

Cell proliferation was assessed by Cell Counting Kit-8 (Dojindo, Kunamoto, Japan) assay, using cellular DNA labeled with the fluorescence reagent. SGC7901 cells in logarithmic phase were inoculated on a 96-well plate at a density of 3×10^4^ cells/well and incubated overnight to allow adherence. Subsequent to washing, the culture medium and IL-8 at fixed concentrations were added to the cells. The cells of each group were incubated for 1–7 days. Nine duplicate wells were employed for each group. At the end of the culture period, WST-8, which produces a water-soluble formazan, was added to the cells. The cells were incubated for an additional 4 h. Colorimetric absorbance was measured by a microplate reader (Multiskan MK3; Thermo Fisher Scientific, Waltham, MA, USA) at 450 nm to obtain an optical density (OD) value. The OD values were calculated using the following equation: OD ultimate value = OD measure value − OD blank value.

### Immunofluorescence staining

A total of 2×10^5^ SGC7901 cells were seeded on a six-well plate and cultured with the fixed concentrations of IL-8 for 72 h. Subsequently, 7×10^4^ cells were placed on coverslips and cultured in RPMI-1640 medium at 37°C to allow adherence. Following fixation in 4% paraformaldehyde for 15 min, a 10-min treatment with 0.5% Triton X-100 (Shanghai Sangon Biotech, Co., Ltd., Shanghai, China) and a 1-h incubation with 4% bovine serum albumin (Wisent Inc., St Bruno, Quebec, Canada) at room temperature, the cells of each group were incubated with PCNA rabbit anti-human monoclonal antibody (Epitomics, Burlingame, CA, USA) at 4°C overnight. Cy3-conjugated affinipure goat polyclonal anti-rabbit IgG (H+L;1:1,000 dilution; Proteintech Group, Wuhan, China) was added for an additional 1-h incubation. The cell nuclei were then labeled with DAPI. The coverslips were analyzed with a laser confocal scanning microscope (LSM710; Zeiss, Oberkochen, Germany).

### Western blot analysis

The cells of each group were incubated for 72 h. The cells were collected and decomposed by 150 μl cell lysis buffer, and the sample was boiled out for 10 min. Subsequent to cooling on ice, the cell lysate was centrifuged for 1 min at 13,201 × g. The supernatant fluid was loaded onto SDS-PAGE (10% separation gel, 5% spacer gel) and electrotransferred to polyvinylidene difluoride film (Bio-Rad, Hercules, CA, USA). The blotted films were placed in blocking solution for 1 h at room temperature. Rabbit anti-human PCNA monoclonal antibody (1:250; Epitomics) was used to probe the blots overnight at 4°C. The film was washed twice and then incubated with goat polyclonal anti-rabbit IgG-horse radish peroxidase secondary antibody (1:1,000; Santa Cruz Biotechnology, Inc., Santa Cruz, CA, USA) for 1 h at room temperature. The film was washed three times and the signal determined by the enhanced chemiluminescence method using an ECL kit (PerkinElmer, Inc., Waltham, MA, USA). The blots were subsequently exposed to plain X-ray film in a darkroom, and the film was scanned by an image analyzer. Grayscale reconstruction was performed using Image J software 1.48 (http://rsb.info.nih.gov./ij/), and the expression rate of PCNA versus that of GAPDH protein, serving as an internal control protein, was calculated. All experiments were repeated three times.

### Reverse transcription quantitative polymerase chain reaction (RT-qPCR) analysis

The cells of each group were inoculated on a six-well plate at a density of 1×10^5^ cells/well and incubated for 72 h. In brief, total RNA of the SGC7901 cells was extracted by TRIzol reagent (Takara, Shiga, Japan) according to the manufacturer’s instructions and reverse-transcribed. RT-qPCR was performed with SYBR Green in a real-time PCR system (Bio-Rad iQ5; Bio-Rad), with each sample analyzed in triplicate. The cycling conditions consisted of one cycle of 95°C for 2 min, 95°C for 15 sec, 60°C for 20 sec and 72°C for 20 sec, and then 40 cycles of 72°C for 30 sec. The primer sequences for the genes analyzed are shown in Table I. The relative levels of PCNA mRNA expression were normalized to those of GAPDH mRNA, and were calculated according to the 2^−ΔΔCt^ method.

### Statistical methods

All data were analyzed using SPSS 13.0 software (SPSS, Inc., Chicago, IL, USA). All results are presented as the mean ± standard deviation. Analysis of variance (ANOVA) of repeated measurement data was used to assess cell proliferation. One-way ANOVA was used to assess protein and mRNA expression levels. The least significant difference method was used to analyze multiple post hoc comparisons. P<0.05 was considered to indicate a statistically significant difference.

## Results

### Effect of IL-8 on SGC7901 cell growth

**Figure 1:**
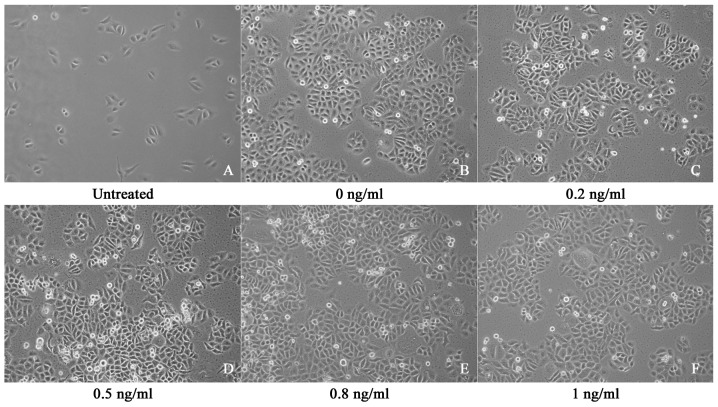
Growth of SGC7901 gastric cancer cells observed by an inverted microscope (magnification, ×10). (A) Prior to IL-8 addition, and (B–F) following the addition of 0, 0.2, 0.5, 0.8 and 1 ng/ml IL-8, respectively, the cells were incubated for 72 h. IL-8, interleukin-8.

The SGC7901 cells proliferated rapidly, exhibiting a fusiform shape, adherence and overlapping growth. The number of cells increased gradually between the first and the sixth days. On the seventh day, cell growth was arrested in the plateau phase with no further increase in the number of cells. The exposure of the cells to IL-8 at concentrations ranging from 0 to 1 ng/ml did not exert a significant effect on growth (Fig. 1).

### Effect of IL-8 on SGC7901 cell proliferation

**Figure 2:**
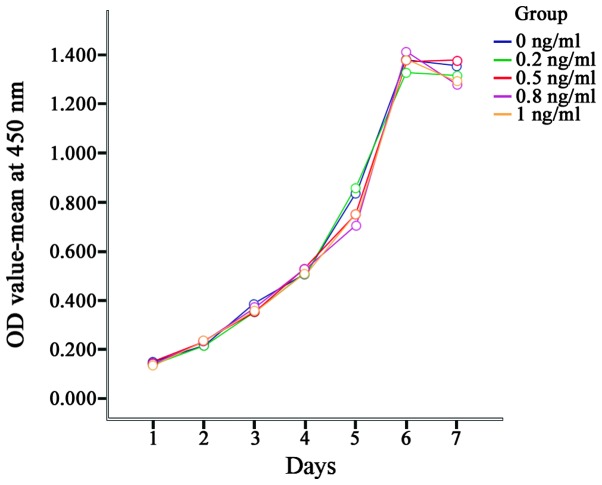
Growth curve of SGC7901 gastric cancer cells exposed to interleukin-8 at concentrations ranging from 0 to 1 ng/ml. OD, optical density.

Between the first and the sixth days, the OD values increased gradually; peak OD values were obtained on the sixth day, with slight decreases on the seventh day. The OD values between the different days were significantly different (P<0.001). However, no significant differences in the OD value between treatment groups were identified (P=0.162). This result indicated that IL-8 exerted no significant effect on gastric cancer cell proliferation (Table II, Fig. 2).

### Effect of IL-8 on PCNA protein expression levels in SGC7901 cells

**Figure 3:**
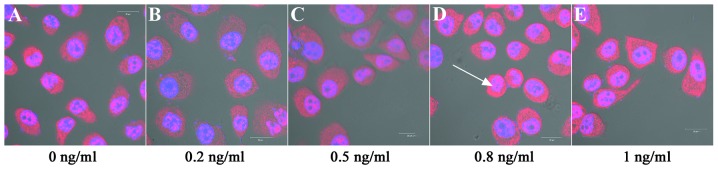
Effect of interleukin-8 on PCNA expression levels in SGC7901 gastric cancer cells (immunofluorescence staining; magnification, ×630). White arrowhead indicates PCNA counterstained with Cy3 (red). PCNA, proliferating cell nuclear antigen.

**Figure 4:**
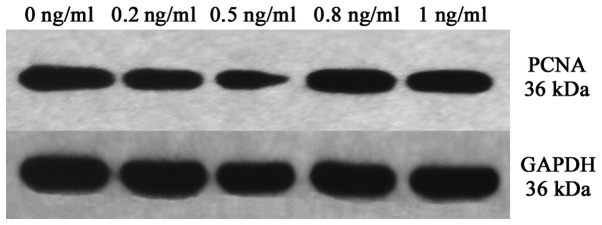
Effect of interleukin-8 on PCNA protein expression levels in SGC7901 gastric cancer cells (western blot). PCNA, proliferating cell nuclear antigen.

**Figure 5:**
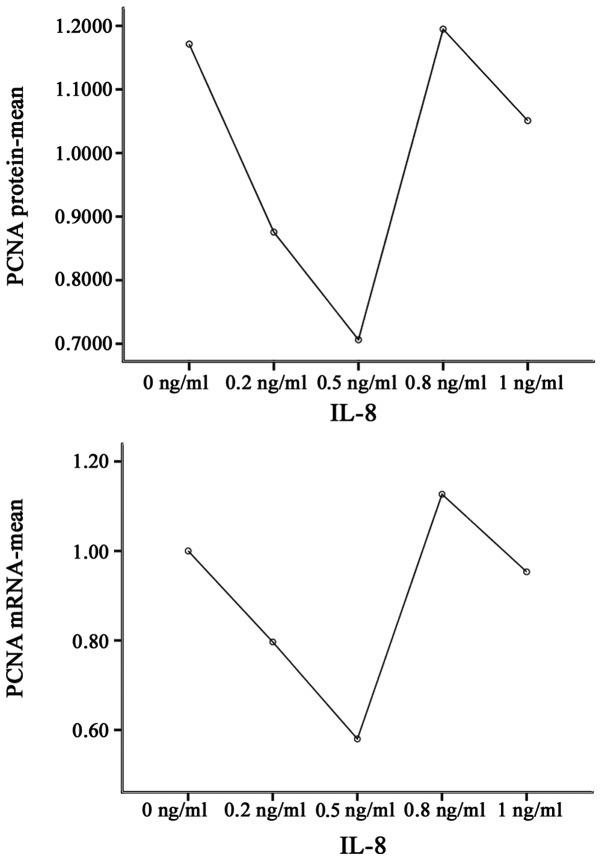
Effect of IL-8 on expression of PCNA protein and mRNA in SGC7901 gastric cancer cells. IL-8, interleukin-8; PCNA, proliferating cell nuclear antigen.

Cell nuclei were detected with DAPI (blue) staining and PCNA protein was counterstained with Cy3-Conjugated Affinipure Goat Anti-Rabbit IgG (red). Fig. 3 reveals that PCNA immunostaining was restricted to the cell cytoplasm and nuclei. Notably, IL-8 significantly affected the PCNA protein expression levels under the experimental conditions compared with the control cells (P<0.001). IL-8 at 0.2, 0.5 and 1 ng/ml concentrations significantly downregulated the expression of PCNA protein, compared with no treatment (P<0.01). By contrast, 0.8 ng/ml IL-8 significantly upregulated the expression levels of PCNA protein (P<0.01; Table III, Figs. 3–5).

### Effect of IL-8 on PCNA mRNA expression levels in SGC7901 cells

**Figure 6:**
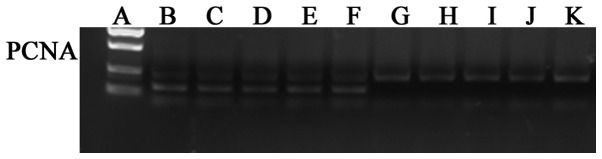
Effect of interleukin-8 on PCNA mRNA expression levels in SGC7901 gastric cancer cells (reverse transcription quantitative polymerase chain reaction). A, marker DL2000 (from top to bottom: 750, 500, 250 and 100 bp; B–F, hGAPDH (0–1 ng/ml, 145 bp); G–K, PCNA (0–1 ng/ml, 229 bp). PCNA, proliferating cell nuclear antigen.

A statistically significant difference between mRNA expression levels in all groups was observed when compared with that of the control group (P<0.001), with PCNA mRNA expression levels exhibiting a similar pattern to the PCNA protein expression levels. IL-8 at concentrations of 0.2 and 0.5 ng/ml significantly downregulated the expression of PCNA mRNA, compared with no treatment (P<0.01). By contrast, treatment with 0.8 ng/ml IL-8 significantly upregulated the expression of PCNA mRNA, compared with no treatment (P<0.05; Table III, Figs. 5 and 6).

## Discussion

Hp, a Gram-negative spiral bacterium, is a predominant stomach pathogen associated with chronic gastric disease that infects >50% of the population worldwide (17). Hp colonizes the human stomach and causes extensive gastric epithelial cell inflammation (10,18). Once Hp adheres to the host gastric epithelial cells, signal transduction is activated through virulence factors, such as cytotoxin-associated antigen (CagA). The inflammatory cascade is immediately initiated, with increased secretion of various inflammatory cytokines, including IL-1, IL-6, IL-8, intercellular adhesion molecule-1, cyclooxygenase-2 and tumor necrosis factor α (19–24). Therefore, infection by CagA-positive Hp is a known risk factor for the development of gastric disease due not only to marked changes in cellular morphology but also the release of cytokines from the gastric epithelium (25).

IL-8 is a multifunctional pro-inflammatory cytokine released through the nuclear factor-κB signaling pathway, which includes extracellular signal-regulated kinase activity (26,27) and mitogen-activated protein kinase (28). IL-8 has been shown, through whole genome analysis, to be the most markedly upregulated gene, and to exert an important role in numerous epithelial cellular responses to Hp infection and in the pathological processes resulting in gastric disease (29). IL-8 production *in vitro* and *in vivo* induced by Hp has been recognized as a host response to microbes. Hp directly increases gastric epithelial IL-8 protein secretion and IL-8 mRNA expression levels (30,31).

Thus far, several studies have identified the association between Hp infection and gastric cancer. Hp infection is known to exert a predominant role in gastric carcinogenesis, a process that commences with chronic gastritis and results in a sequence of atrophic gastritis, metaplasia, dysplasia and subsequently, gastric cancer (32,33). Therefore, WHO classified Hp as a group I carcinogen in 1994 (34).

Increased IL-8 expression levels have been detected in numerous types of cancer cell, suggesting that IL-8 may function as a significant regulatory factor within the tumor microenvironment (35). As the overexpression of IL-8 is induced by Hp infection, IL-8 has been associated with gastric cancer. *In vitro*, IL-8 is produced by gastric cancer cells in response to exposure to the cytotoxic strain of Hp (36), and IL-8 is involved in the progressive growth of gastric cancer by autocrine or paracrine mechanisms (8). *In vivo*, IL-8 produced by gastric tumor cells may regulate the neovascularization, growth and spread of human gastric cancer (37). IL-8 levels have also been significantly correlated with depth of invasion, venous invasion and lymphatic invasion, and may be an independent prognostic factor in human gastric carcinomas (38). In a previous study using recombinant IL-8 treatment, IL-8 was reported to be associated with the adhesion, migration and invasion of SGC-7901 human gastric cancer cells (39). Similarly, using cDNA and small interfering (si)RNA transfectants, Kuai *et al* (9) reported that IL-8 was essential in human gastric cancer cell adhesion, migration, invasion and chemosensitivity.

Cell proliferation is a key process in the growth of carcinoma. Several recent studies have indicated that upregulated IL-8 mediates tumorigenic and mitogenic effects (40), and stimulates proliferation in a variety of human cancer cell types, including human melanoma (41), squamous cell carcinoma (42,43), ovarian cancer (44), non-small cell lung cancer (45) and colon cancer cells (46). By contrast, downregulation of IL-8 via siRNA inhibited proliferation and delayed G1 to S phase cell cycle progression in several types of cancer, including ER-negative breast cancer (47). However, whether IL-8 influences cell proliferation of gastric cancer remains unclear. As determined by the associations amongst IL-8, Hp and gastric cancer, IL-8 was hypothesized to promote cell proliferation in gastric cancer. The present study aimed to determine the effects of IL-8 on gastric cancer cell proliferation. A previous study indicated that treatment of SGC7901 cells with recombinant IL-8 at concentrations ranging between 0 and 100 ng/ml did not exert a significant effect on cancer cell proliferation (39). Similarly, using cDNA and siRNA transfectants, overexpression of IL-8 in the MKN-45 gastric cancer cell line and silencing IL-8 expression in the KATO-III gastric cancer cell line was not significantly associated with cell proliferation (9). The IL-8 level produced by gastric cancer cells is marginal. For example, *in vitro*, the highest levels of IL-8 were 0.17 ng/ml in the IM95 gastric cancer line cultured for three days (48). Therefore, the effect of IL-8 on gastric cancer cell proliferation may have been associated with IL-8 dosage. However, in the present study, the proliferation rate of SGC7901 cells treated with IL-8 at concentrations ranging between 0 and 1 ng/ml was not significantly different from that of the control cells. This result indicated that IL-8 exerted no significant effect on the proliferation of SGC7901 gastric cancer cells, although IL-8 was considered to promote invasion, migration and adhesion of gastric cancer cells (9). In previous studies, no significant effect of IL-8 on cell proliferation in several types of hepatocellular and breast cancer cells was observed (49,50).

PCNA, an auxiliary protein of DNA polymerase δ located in the nuclei of tumor cells, is known as a cell cycle-related nuclear antigen and is synthesized in late G1 and S phase. PCNA levels therefore correlate with the cell proliferative state (51,52). Although no effect of IL-8 on the proliferation of SGC7901 gastric cancer cells was identified in the present study, notably, the data revealed that PCNA expression levels were associated with IL-8. Immunofluorescence staining and western blot analysis were used to observe the expression levels of PCNA protein, and the qPCR method was employed to assay the levels of PCNA mRNA. The data demonstrated that IL-8 had a significant effect on PCNA protein and mRNA expression levels in the SGC7901 cells, in a dose-dependent manner. At a 0.8-ng/ml dosage, IL-8 significantly increased the expression levels of PCNA protein and mRNA. However, IL-8 significantly inhibited the expression of PCNA at the other dosages. This may be associated with the IL-8 regulatory mechanism of PCNA expression, however, this regulatory effect does not appear to be involved in gastric cancer cell proliferation. Other potential regulatory mechanisms of IL-8 in gastric cancer require investigation.

In conclusion, the present study demonstrated that IL-8 exerts no direct effect on the proliferation of gastric cancer cells, but influences the expression levels of PCNA protein and mRNA, depending on the IL-8 dosage. The findings suggest that IL-8 is a potent pro-inflammatory cytokine with multiple effects on the development of gastric cancer.

## Figures and Tables

**Table I:** Primer sequences used for quantitative polymerase chain reaction.

mRNA	Sense primer sequence	bp
hGAPDH-F	5′-GGGTGTGAACCATGAGAAGTATG-3′	145
hGAPDH-R	5′-GATGGCATGGACTGTGGTCAT-3′	
PCNA-F	5′-TCATTACACTAAGGGCCGAAGA-3′	229
PCNA-R	5′-GCACAGGAAATTACAACAGCATC-3′	

F, forward; R, reverse; PCNA, proliferating cell nuclear antigen.

**Table II:** Effect of interleukin-8 on SGC7901 gastric cancer cell proliferation (optical density).

Group	Day 1	Day 2	Day 3	Day 4	Day 5	Day 6	Day 7
0 ng/ml	0.150±0.052	0.216±0.012	0.387±0.060	0.507±0.017	0.838±0.024	1.381±0.019	1.356±0.030
0.2 ng/ml	0.141±0.001	0.214±0.012	0.352±0.020	0.514±0.006	0.860±0.016	1.331±0.060	1.318±0.047
0.5 ng/ml	0.148±0.020	0.233±0.006	0.354±0.001	0.530±0.035	0.751±0.036	1.371±0.064	1.380±0.003
0.8 ng/ml	0.139±0.005	0.232±0.016	0.368±0.009	0.531±0.035	0.705±0.016	1.414±0.042	1.281±0.033
1 ng/ml	0.133±0.007	0.236±0.010	0.354±0.006	0.512±0.024	0.752±0.046	1.386±0.034	1.293±0.039

**Table III:** Effect of interleukin-8 on the expression levels of PCNA protein and mRNA in SGC7901 gastric cancer cells.

Group	PCNA protein	PCNA mRNA
0 ng/ml	1.171±0.003	1.00±0.09
0.2 ng/ml	0.876±0.006^b^	0.80±0.02^b^
0.5 ng/ml	0.706±0.011^b^,^c^	0.58±0.03^b^,^c^
0.8 ng/ml	1.195±0.006^b^,^c^,^d^	1.13±0.06^a^,^c^,^d^
1 ng/ml	1.051±0.001^b^,^c^,^d^,^e^	0.95±0.06^c^,^d^,^e^

a P<0.05 and

b P<0.01, vs. 0 ng/ml;

c P<0.01, vs. 0.2 ng/ml;

d P<0.01, vs. 0.5 ng/ml;

e P<0.01, vs. 0.8 ng/ml.

PCNA, proliferating cell nuclear antigen.
